# Enhanced neural plasticity of the primary visual cortex in visual snow syndrome: evidence from magnetoencephalographic gamma oscillations

**DOI:** 10.1093/braincomms/fcaf505

**Published:** 2025-12-24

**Authors:** Elena V Orekhova, Anna M Plieva, Sophia M Naumova, Tatiana S Obukhova, Andrey O Prokofyev, Anastasiia V Petrokovskaia, Ada R Artemenko, Tatiana A Stroganova

**Affiliations:** Center for Neurocognitive Research (MEG Center), Moscow State University of Psychology and Education, Moscow, Russia; Center for Neurocognitive Research (MEG Center), Moscow State University of Psychology and Education, Moscow, Russia; National Research University Higher School of Economics, Moscow, Russia; Center for Neurocognitive Research (MEG Center), Moscow State University of Psychology and Education, Moscow, Russia; Center for Neurocognitive Research (MEG Center), Moscow State University of Psychology and Education, Moscow, Russia; Loginov Moscow Clinical Scientific Center, Moscow, Russia; Sechenov First Moscow State Medical University of the Ministry of Health of the Russian Federation (Sechenov University), Moscow, Russia; Center for Neurocognitive Research (MEG Center), Moscow State University of Psychology and Education, Moscow, Russia

**Keywords:** visual snow syndrome, repetition-related plasticity, magnetoencephalography, gamma oscillations, excitation–inhibition balance

## Abstract

Visual snow syndrome is a neurological disorder characterized by persistent visual disturbances and associated symptoms. Although the neural basis of the visual snow syndrome remains poorly understood, it may involve increased neuronal excitability and/or altered neuroplasticity in the visual cortex, which could, in turn, affect visual gamma oscillations. An altered excitation-inhibition balance is hypothesized to alter the modulation of gamma power and frequency by stimulation intensity, while maladaptive neuroplasticity may impact time-dependent changes in gamma power during repeated stimulation. To investigate potential alterations in the excitation–inhibition balance and neuroplasticity in visual snow syndrome, we used magnetoencephalography to record visual gamma oscillations in 26 patients with this disorder and 27 healthy controls. Participants were exposed to repeatedly presented high-contrast annular gratings, which were either static or drifting at varying speeds to systematically manipulate stimulation intensity. We also assessed heart rate variability during rest and repetitive visual stimulation to explore the relationship between time-dependent gamma changes and parasympathetic activation, which is known to promote activity-dependent plasticity. Our results showed no significant group differences in gamma power or frequency, nor in their modulation by drift rate, suggesting that the excitation–inhibition balance in the V1 area remains largely intact in visual snow syndrome. Both groups exhibited an initial brief decrease in gamma power followed by a sustained linear increase with stimulus repetition, likely reflecting activity-dependent plasticity. Heart rate variability parameters were comparable across groups, with the parasympathetic–sympathetic balance index correlating with repetition-related increase in gamma power, further supporting the link between time-dependent gamma changes and neuroplasticity. Notably, patients with visual snow syndrome exhibited a steeper repetition-related increase in gamma power, indicating atypically heightened activity-dependent plasticity in this group. These findings provide the first experimental evidence suggesting that altered activity-dependent neuroplasticity plays a role in the pathophysiology of the visual snow syndrome. Furthermore, they identify repetition-related increases in gamma power as a potential biomarker of aberrant neuroplasticity, offering novel insights into the pathophysiology of the visual snow syndrome and potential avenues for targeted therapeutic interventions.

## Teaser

Orekhova *et al*. investigated gamma oscillatory responses to repeated visual stimuli to assess short-term plasticity in the visual cortex of patients with visual snow syndrome. Compared to controls, patients exhibited a more pronounced increase in gamma power with stimulus repetition, suggesting abnormally enhanced activity-dependent plasticity.

## Graphical Abstract

**Graphical Abstract:**
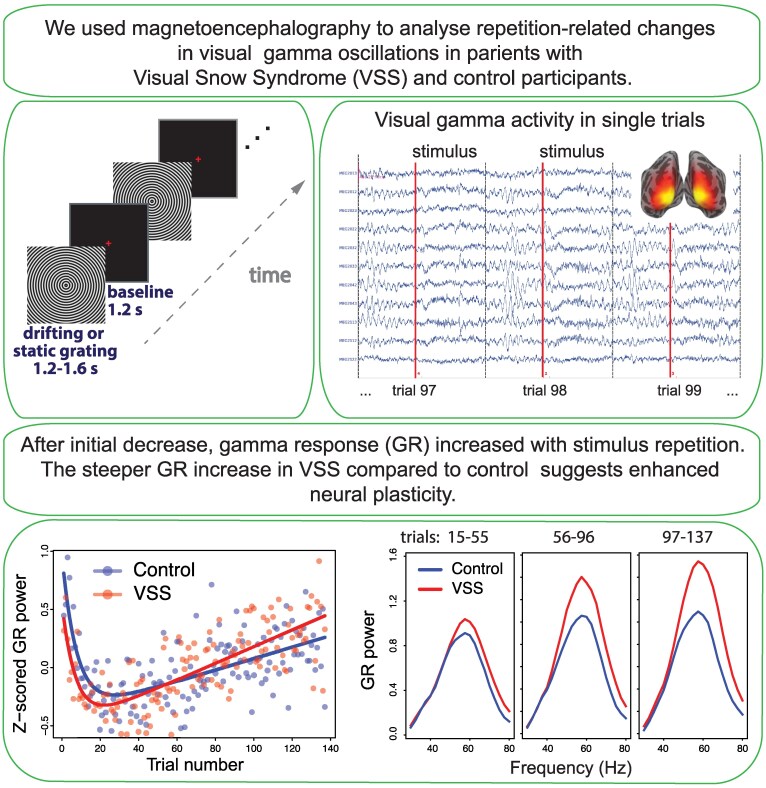

## Introduction

Visual snow syndrome (VSS) is a neurological disorder marked by the continuous presence of small, flickering dots throughout the entire visual field, resembling white noise on an improperly tuned television.^1^ In addition to this visual ‘snow’, individuals with VSS often experience other visual disturbances, such as photophobia, palinopsia, nyctalopia, and non-visual symptoms (e.g. migraine, tinnitus, trouble concentrating, fatigue, and anxiety).^2-5^

While the exact causes of VSS are unknown, it is widely hypothesized to be related to neural hyperexcitability in visual cortical networks.^6,7^ It remains unclear, however, whether this hyperexcitability is linked to a deficit in inhibition.^8^ Additionally, the degree of involvement of the primary visual cortex (V1) in VSS is uncertain. While many studies emphasize the role of areas downstream of V1, such as the lingual gyrus^9-11^ or higher-order supramodal associative areas,^12^ there is also some evidence for structural^1,13^ and functional^7,14-16^ abnormalities in V1. Detecting the earliest affected cortical level would enhance our understanding of the VSS mechanisms.

Investigating narrow-band visual gamma oscillations can help clarify how changes in neural excitability in V1 contribute to the pathophysiology of VSS. These high-frequency oscillations (40–90 Hz) are generated in V1^17,18^ in response to stimuli of certain characteristics, such as high-contrast gratings, especially those moving with a certain ‘optimal’ speed,^19^ and can be reliably recorded with MEG. Animal studies have shown that gamma oscillations are generated through recurrent interaction between cortical inhibitory and excitatory neurons, with significant imbalances in their activity influencing the frequency and power of gamma oscillations (e.g.^20-22^). The only MEG study on patients with VSS reported increased gamma power in response to static high-contrast gratings, which was interpreted as evidence of heightened neural excitability.^14^ Here, we sought to replicate this result as well as investigate other important properties of gamma oscillations that, as described below, may shed light on the excitation-inhibition (E–I) balance and neuroplasticity of visual cortex.

It has been previously demonstrated that increasing the drift rate of a visual grating—a proxy for excitatory drive—induces a bell-shaped change in gamma power.^19,23^ This non-linear modulation likely reflects the progressive recruitment of inhibitory neurons, which initially enhance gamma oscillations but eventually disrupt gamma synchronization at excessively high excitatory drive.^24,25^ In case of deficient inhibition, this modulation curve may be altered, shifting the optimal excitatory drive for gamma generation towards higher values. Such alterations in gamma modulation have been observed in neuropsychiatric disorders, aligning with other evidence of reduced inhibitory function in V1 circuitry.^23,26^ The ***first aim*** of this study was to apply this approach to VSS to assess whether potentially increased neural excitability in V1 in this disorder is associated with weakened inhibition.

Beyond altered neural excitability, changes in neural plasticity have been proposed as putative pathophysiological mechanisms underlying both the symptoms and the functional and structural alterations in visual and extra-visual areas in VSS.^27^ Neural plasticity refers to synaptic modifications in network-level interactions, which, in case of the Hebbian plasticity, are driven by prior neural activity, enabling the neural network to adapt its stimulus processing based on sensory experience.^28^ While maladaptive synaptic plasticity has been clearly implicated in ‘phantom sensations’ in somatosensory (e.g. chronic pain) and auditory (e.g. tinnitus) domains,^29-32^ its role in VSS symptoms is unclear. To date, this hypothesis remains speculative, as there is a lack of experimental studies investigating neural plasticity in VSS.

Recent studies demonstrate that visual gamma oscillations recorded by MEG offer a means to investigate neuroplasticity induced by stimulus repetition.^33^ While a repetition of an identical visual stimulus typically results in decreased neuronal firing rates,^33,34^ and reduction of evoked responses in EEG/MEG,^35-37^ it is also associated with an increase in gamma response power and frequency.^33,36^ These repetition-related gamma changes are stimulus-specific and can persist for tens of minutes after the exposure ends. Animal studies have shown that the stimulus-specific increases in gamma power likely reflect Hebbian plasticity, which reshapes receptive fields in V1 for frequently presented stimulus, thereby supporting implicit perceptual learning.^33,38^ Our ***second aim*** was to investigate, for the first time, potential alterations in neural plasticity in VSS by examining repetition-related changes in visual gamma parameters in this population compared to healthy controls. We also examined the concomitant repetition-related changes in MEG evoked responses, given their proposed link to impaired habituation in patients with VSS.^7,15,16^

Hebbian plasticity in cortical circuits is enhanced during states of arousal and attention, processes associated with the release of neuromodulators^39,40^ that facilitate plasticity^41^ and are linked to shifts in sympathetic-parasympathetic balance.^42,43^ In individuals with VSS, parasympathetic activity may differ from that of controls, potentially due to the frequent presence of comorbid psychiatric symptoms such as anxiety and depression.^44^ Therefore, our ***third aim*** was to examine the role of autonomic regulation in the gamma-derived measure of V1 neuroplasticity in both neurotypical individuals and those with VSS. To this end, we assessed heart rate variability in relation to repetition-related changes in visual gamma responses.

In summary, the primary focus of the present study was to test the hypotheses of an elevated *E–I* ratio and altered Hebbian neuroplasticity in the V1 of patients with VSS. To address these hypotheses, we investigated changes in gamma oscillations associated with variations in excitatory input to the visual cortex, as well as their time-dependent modulation in response to repeated stimuli.

## Materials and methods

### Participants

Data were collected from 26 patients diagnosed with VSS and 27 age- and gender-matched neurologically healthy control subjects between 2020 and 2024. The sample size was chosen based on the results of a previous study that reported increased average visual GR power in participants with VSS relative to healthy controls with effect size *d* = 0.74.^14^ With our sample size, we expected to detect this effect with 84% power at *P* = 0.05.

Patients were recruited from an online VSS community and through clinic referrals. All patients underwent neurological examination by a board-certified neurologist and were diagnosed with VSS in accordance with relevant diagnostic criteria.^45^ Information about their neurological and visual symptoms is presented in Tables 1 and 2. Thirteen subjects from the patient group reported visual snow (VS) from early childhood (before age 10), while the other 13 developed VS after age 14. Subjects who developed VS due to substance abuse were excluded from the study. Two patients were taking antidepressants: one on venlafaxine (75 mg/day) and another on escitalopram (5 mg/day); both had been taking their respective medications for more than three months. The other 24 participants were medication-free.

**Table 1:** Characteristics of the participants

	VSS, mean+/−SD	Control mean+/−SD	*t*, *P*
*N*	26 (14 males)	27 (16 males)	
Age	26.7 +/− 5.6	26.9 +/− 4.2	n.s.
Migraine	20 (77%)	0%	
Migraine with aura	7 (27%)	0%	
Tinnitus	14 (54%)	0%	
STAI (all life)^a^	51.4 +/− 9.5	40.1 +/− 9.6	t(51) = 4.3, *P* = 0.00008
Visual discomfort score^b^	25.9 +/− 13.1	8.5 +/− 7.9	t(50) = 5.8, *P* < 0.00001

STAI—State-Trait Anxiety Inventory^46^; Visual discomfort was assessed using the Visual Discomfort Scale^47^.

^a^Information was not available for one VSS and one control participants.

^b^Information was not available for two VSS and one control participants.

**Table 2:** Percentage of patients who experienced visual phenomena commonly associated with VSS

Visual symptoms^a^	*N* (%)
Palinopsia (I): afterimages	15 (58%)
Palinopsia (II): trailing of moving objects	10 (38%)
Enhanced entopic phenomena	19 (73%)
Photophobia	19 (73%)
Impaired night vision (nyctalopia)	15 (58%)

^a^All but one patient with VSS rated their visual symptoms on a 5-point Likert scale (1—absence of the symptom, 2—very rare, 3—rare, 4—often, 5—all the time). The table lists the number of patients who reported ‘4’ or ‘5’ on the respective scales.

The ethics committee of the Moscow State University Psychology and Education approved the study and all subjects gave written informed consent.

### Experimental paradigm

In all VSS participants, the recording session began with a 5-minute period of rest with eyes closed, followed by a 5-minute period with eyes open. The resting state data were available in 22 of 27 control participants. The ‘eyes closed’ condition was not analysed in this study, whereas data from the ‘eyes open’ condition were used to estimate resting-state heart rate variability (HRV).

**Figure 1:**
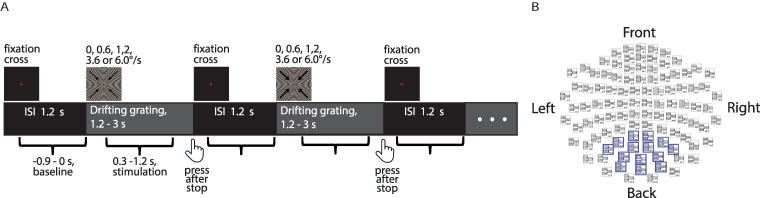
**Design of the experiment. A.** Visual stimulation paradigm. Curly brackets indicate 0.9-second intervals included in the time-frequency analysis. **B.** MEG sensor layout. Blue boxes highlight locations from which gradiometers with the strongest gamma response were selected for sensor-level analysis. ISI—inter-stimulus interval.

During the visual task (Fig. 1A) presented after the resting condition, participants observed a sequence of annular gratings (18˚, full contrast, spatial frequency 1.66 cycles/degree) that either drifted towards the centre at one of four velocities (0.6˚/s, 1.2˚/s, 3.6˚/s, or 6.0˚/s) or remained static. Eye-tracking was not performed during MEG acquisition. Although participants were instructed to maintain fixation, the absence of direct eye-movement monitoring limits our ability to assess potential contributions of eye-related measures to trial-wise gamma dynamics.

All trials started with a fixed 1.2 s prestimulus interval during which a fixation cross was presented in the centre of the screen. The presentation time for each stimulus ranged randomly from 1.2 to 1.6 s. Participants were instructed to press a button as soon as the stimulus disappeared. The new trial started immediately after the button press. If the button was not pressed within 1 s after the stimulus disappearance, the trial was considered a miss, and the message ‘You're late!’ was displayed to the participant for 1.0 s. The responses earlier than 150 ms after stimulus disappearance were considered commission error trials. To reduce fatigue and boredom, participants were shown 3–6 s cartoon animations after every 5–10 gratings. For each participant, 90 gratings of each type were presented.

Subjects were offered a break in the middle of the session. There was no significant difference between the groups in the duration of the first session (Mann–Whitney U-test, *Z* = 0.97, Median_VSS_ = 254 trials, Median_Control_ = 258 trials, *P* = 0.33) or in the break length (Mann–Whitney U-test, *Z* = 0.64, Median_VSS_ = 41 s, Median_Control_ = 38 s, *P* = 0.33). For all participants, data uninterrupted by breaks were available for the first 1 to 137 trials. The number of artefact-free trials did not differ between groups, either for the 1–137 trial interval (mean: *N*_Control_ = 132, *N*_vss_ = 131, Wilcoxon rank sum test: *Z* = 1.69, *P* = 0.09) or for the entire experiment (mean *N*_Control_ = 421, *N*_vss_ = 412, *Z* = 1.40, *P* = 0.17). Excluded trials were approximately evenly distributed over time (Supplementary Fig. 1).

### Recording and pre-processing of electrophysiological data

The MEG data were recorded at the Moscow Centre for Neurocognitive Research using an Elekta VectorView Neuromag 306-channel MEG detector array (Helsinki, Finland). Prior to MEG recording, each participant’s individual head shape was digitized using an electromagnetic position and orientation tracking system (FASTRAK, Polhemus). This process involved recording three anatomical landmarks (nasion, left and right preauricular points), four head position indicators (HPI) coils, and >100 additional points evenly distributed over the head surface.

During data acquisition, the positions of the HPI coils were continuously tracked at a 5 Hz sampling rate (continuous HPI), providing real-time information on the participant’s head position within the MEG helmet.

The MEG signal was acquired with 0.1 Hz high-pass and 330 Hz low-pass built-in filters and sampled at 1000 Hz. To monitor heartbeats, electrocardiogram (ECG) electrodes were placed on the sternum and the midaxillary line (ECG lead V6).

The MEG signal was subsequently processed using MaxFilter software (version 2.2) to minimize interference from external artefact sources through the temporal signal-space separation (tSSS) method^48^ and to provide motion correction. As part of the MaxFilter processing, the data were transformed to a standard head position (*x* = 0 mm; *y* = 0 mm; *z* = 40 mm) across all experimental blocks. Further data preprocessing steps were performed with the MNE-python toolbox (v.1.7.1) and described in the Supplementary materials.

### MEG data analysis

#### Gamma oscillations: mean power and frequency of gamma response (GR)

Since not all our participants underwent structural MRI of the brain, we performed the main analysis in the sensor space. Only data from planar gradiometers were used for sensor-level analysis. Gamma response (GR) power was analysed in 0.3–1.2 s interval following stimulation onset, and −0.9–0.0 s interval was used as a baseline. To isolate stimulus-related induced activity, the averaged evoked responses were subtracted from single-trial data according to stimulus type. Time-frequency analysis was performed using the multitaper method with a bandwidth of 10 Hz, a time step of 2 ms, and a frequency resolution of 2.5 Hz. We estimated normalized response power as (stimulation−baseline)/baseline * 100%. We then averaged spectra across sensors (1–4 sensors) where the average normalized power in the 35–80 Hz range exceeded 80% of that in the ‘maximal sensor’. To minimize the contribution of noise, these sensors were selected from the posterior set of gradiometers (Fig. 1B), where maximal GR is typically expected.^49^

To estimate the subject's mean weighted GR power and frequency, we used the approach employed in our previous studies (e.g.^23^). Spectra were averaged for all artefact-free epochs, separately for drift rate conditions. The GR power was then calculated as the average of those spectrum values that exceeded 2/3 of the peak value in the frequency range 35–100 Hz. The GR frequency was estimated as the centre of gravity of the spectrum values used to calculate the GR power.

To ensure robustness of the findings, we also repeated analysis of gamma oscillations in the source space using a linearly constrained minimum variance (LCMV) beamformer approach. When available, individual subjects MRIs were used; otherwise, a template brain warped to the subject’s digitized head shape via the recently developed ‘pseudo-MRI’ method (Jaiswal *et al*., 2025) was employed. The details on the source-level analysis methods and the respective results are provided in the Supplementary materials, including Supplementary Fig. 4 and Supplementary Table 3.

#### Gamma oscillations: single-trial GR power and frequency

For the single-trial analysis, GR power and frequency were estimated separately for each trial. The single-trial power was averaged over a range of ±15 Hz relative to the average peak frequency specific for the subject and condition. The single-trial frequency was calculated as the frequency weighted by the GR power in this frequency range.

#### Single-trial analysis of event-related fields (ERF)

Because some previous studies^7,15,16^ reported reduced habituation of event-related responses in VSS, we also examined in our participants the time-dependent changes in event-related fields (ERF) evoked by the appearance of the gratings. The methods of ERF analysis are described in the Supplementary materials.

### Heart rate variability

HRV was assessed using the Systole v0.2.4 package.^50^ Two periods of data were analysed: (1) a 5-minute rest period with eyes open and (2) the first block of the experiment, before the break. ECG data were processed to identify R-peaks using a moving average algorithm. Artefacts such as missed/additional peaks or ectopic beats were corrected using the ‘correct_rr’ function. ECG parameters assessed included heart rate in beats per minute (BPM) and basic HRV metrics (Supplementary materials). We then calculated HF power in normalized units: HFnu = HF/(HF + LF)*100, where HF and LF are the spectral power of R-R intervals in 0.15–0.40 and 0.04–0.15 Hz ranges, respectively. HFnu accounts for individual and contextual differences in overall heart rate variability and allows assessment of the relative contribution of parasympathetic activity to autonomic balance, regardless of the absolute BMP or HRV levels.^51,52^

### Statistical analysis

ANOVA was performed using Statistica software (StatSoft, Inc., STATISTICA, v. 12). A mixed ANOVA with Group and Condition factors (drift velocities: 0, 0.6, 1.2, 3.6, and 6.0°/s) and normalized age as covariance was used to analyse group differences in mean GR power and frequency. GR power values were log-transformed to normalize their distributions. Greenhouse-Geisser (G–G) correction was applied to correct for violation of the sphericity assumption. Partial eta squared (*ηp*2) was used to estimate ANOVA effect sizes. The same ANOVA design was used to compare group differences in reaction time. The Wilcoxon rank-sum test was used to compare group differences in error rates.

To analyse single-trial GR power, we used the first 137 epochs, which were available for all participants without interruption. For each subject, trials with extremely high or low GR power were excluded based on the interquartile range approach,^53^ with a multiplication coefficient of 2. To estimate general patterns of repetition-related changes, single-trial GR values were z-transformed separately for each subject and stimulus type and then averaged across all subjects and stimulus types according to the trial order number. The resulting average was then visually inspected for trends.

Because the averaged z-transformed GR power exhibited the clear biphasic changes described previously for the repetition of static stimuli,^36^ we followed the strategy used by the previous authors and fitted the time course of GR power as the sum of exponential and linear processes over the trial number: y ∼ A * exp(−trialN/tau) + B * trialN + C, where tau is the time constant of early gamma decrease. To determine the inflection point, we also fitted a broken line model to the same data using the ‘segmented’ package in R. We further analysed only repetition-related changes after the inflection point. The time courses of GR frequency were fitted using linear functions.

Linear mixed model (LMM) analysis, implemented using the ‘lme4’ package in R, was employed to assess factors affecting variations in ‘raw’ (i.e. not z-scored) GR power: trial order number (trialN) and group (1st group: VSS, 2nd group: control). Random factors (subject and condition) were included with both intercepts and slopes when feasible and appropriate. Model comparison and selection were based on the Akaike information criterion (AIC), Bayesian information criterion (BIC), and log-likelihood (logLik) test. The effects of trial order number were estimated from beta-coefficients as the change in the dependent variable (GR power or frequency) per 100 trials. Confidence intervals for LMM regression coefficients were estimated via bootstrapping using the ‘bootstrap_parameters’ function from the R package ‘parameters’.

To quantify individual changes in GR power across trials, we employed a linear regression model using z-transformed values (z_score ∼ trialN) for each drift rate condition. The resulting coefficients were averaged across conditions per subject, yielding a personalized metric of repetition-related changes in GR power.

The Spearman method was used for correlation analysis. The Mann–Whitney U-test was used for group comparison of HRV metrics due to their non-Gaussian distribution. Student's two-sample *t*-test was used for group comparison of normally distributed data.

## Results

### Behavioural performance

Reaction time (RT) data were available for 25 of 26 VSS and 24 of 27 control participants, with 4 subjects’ data lost due to technical issues. Omission error rates were comparable between groups (Median_VSS_ = 0.24%, Median_Control_ = 0.48%; Wilcoxon *W* = 240, *P* = 0.23, Cohen’s *d* = 0.35). The rate of commission errors was significantly higher in the VSS group compared to controls (Median_VSS_ = 3.54%, Median_Control_1.80%; Wilcoxon *W* = 414, *P* = 0.023, Cohen’s *d* = 0.69). This suggests that while both groups showed similar task engagement, VSS participants exhibited greater difficulty in response inhibition.

ANOVA revealed a significant effect of Condition on RT (*F*(4188) = 10.33, G–G *ε* = 0.47, *P* = 0.0001, *ηp*² = 0.18), with RT decreasing as drift rate increased. However, neither Group effect (*F*(2,47) = 0.28, *P* = 0.59, *ηp*² = 0.01) nor Group*Condition interaction (*F*(4188) = 1.53, G–G *ε* = 0.47, *P* = 0.20, *ηp*² = 0.03) reached significance. Thus, while drift rate significantly influenced RTs, the performance pattern was similar across groups.

### No group differences in grand average gamma power, frequency, or their modulation by drift rate

Given a statistical power of 80% and an alpha level of 0.05, the minimal detectable partial eta squared (*ηp*²) for our analyses was between 0.09 and 0.13. This indicates the study was sufficiently powered to detect medium-to-large effects, though small effects may have gone undetected.

**Figure 2:**
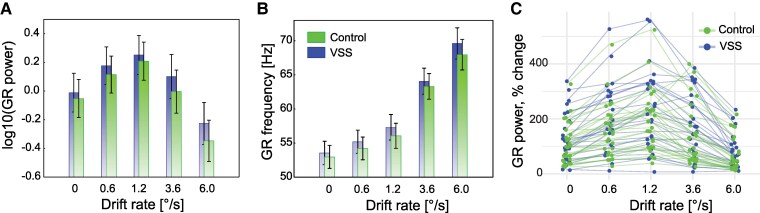
**Parameters of the grand average gamma response (GR). A, B.** GR spectra were averaged across all artefact-free trials for drift rate conditions. Shown are log-transformed average GR power (**A**) and average GR frequency (**B**) in participants with VSS and controls. Vertical bars denote 0.95% confidence intervals; mixed ANOVA revealed no significant effect of Group or Group*Condition interaction. **C.** Interindividual variability of GR power.

ANOVA analysis of the average GR power revealed a robust effect of condition (*F*(4,200) = 81.0, G–G *ε* = 0.43, *P* < 1e−6, *ηp*^2^ = 0.62; Fig. 2A). In accordance with previous results,^19^ an average GR power increased with the drift rate, peaking at 1.2°/s, before declining as the drift rate exceeded this value. Neither Group effect (*F*(1,50) = 0.68, *P* = 0.41, *ηp*^2^ = 0.01) nor Group*Condition interaction (*F*(4,200) = 0.69, G–G *ε* = 0.43, *P* = 0.6, *ηp*^2^ = 0.01) reached significance. Notably, there was a substantial inter-individual variability in GR power (Fig. 2C).

For the average GR frequency, ANOVA also showed a highly significant effect of Condition (*F*(4,200) = 325.9, G–G *ε* = 0.37, *P* < 1e−6, *ηp*^2^ = 0.86; Fig. 2C), but no effects of Group (*F*(1,50) = 0.81, *P* = 0.37, *ηp*^2^ = 0.02) or Group*Condition interaction (*F*(4,200) = 0.32, *P* = 0.86, *ηp*^2^ = 0.01). In both groups, mean GR frequency consistently increased with the drift rate, reaching its maximum at 6.0°/s. Gamma frequency significantly decreased with age (*F*(1,50) = 5.24, *P* = 0.026, *ηp*^2^ = 0.09), aligning with previous findings.^19,54^

The ANOVA results for GR parameters estimated in source space were essentially identical to those obtained from GR parameters measured at the gradiometer sensors (see Supplementary materials for the source space ANOVA results).

### Repetition-related increase in GR power is more pronounced in VSS participants than in controls

**Figure 3:**
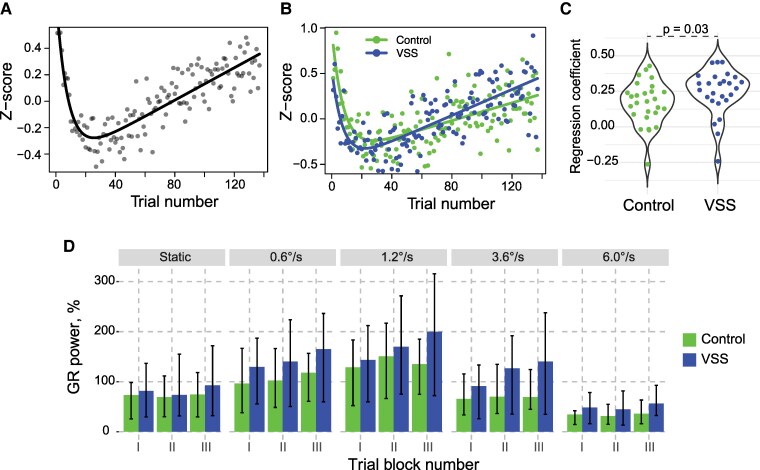
**Repetition-related changes of gamma response (GR) power. A.** Time course of z-transformed GR power across all participants. **B.** Group-specific time courses of z-scored GR power (VSS versus controls). **C.** Violin plots showing individual regression coefficients calculated for trials 15–137 and averaged over drift rates in the VSS and control groups. Group comparison used Mann–Whitney U tests. **D.** Median values of GR power in three sequential blocks of trials (I: 15–55, II: 56–96, III: 97–137) plotted separately for experimental groups and drift rate conditions. Whiskers mark 75% percentile-based confidence intervals.

Analysis of the *z*-transformed GR power averaged over all participants according to the trial number revealed a distinct biphasic pattern: a rapid initial decrease followed by a near-linear increase. Figure 3A illustrates this pattern over 137 trials, modelled as a combination of exponential decay and linear increase (*R*²adj = 0.74). The early decrease exhibited a time constant tau = 7.8 repetitions (CI95% = [6.0, 9.6]). Separate analyses for VSS (*R*²adj_VSS_ = 0.54, tau_VSS_ = 7.3, CI95%_VSS_ = [3.7, 10.7]) and control groups (*R*²adj_control_ = 0.40, tau_control_ = 7.7, CI95%_control_ = [4.7, 10.7]) demonstrated similar biphasic patterns (Fig. 3B). A broken-line fit estimated the inflection point at 14.6 trials (CI95% = [12.6, 16.6]). Given our focus on the later repetition effect, potentially linked to Hebbian plasticity,^33,36^ we subsequently limited the analysis to trials 15–137.

Since the single trial GR power was better explained by the trial number than by the number of stimulus-specific repetition (Supplementary materials), we fitted the following LMM to the data:

GR_power ∼ trialN * group + (1 + trialN | subject) + (1 + trialN | condition), where trialN is the trial order number.

Analysis revealed a large and highly significant trialN effect: *t*(22.00) = 5.03, *P* < 0.0001. There was also significant trialN × group interaction: *t*(51.09)= −2.26, *P* = 0.028 due to a steeper GR power increase with trial number in participants with VSS. In patients with VSS, GR power increased by 0.45 units per 100 trials (CI_95%_ = [0.28 0.60]), while in control participants the increase was 0.19 units (CI_95%_ = [0.05 0.34]). Note that these values represent 45% and 19% increase relative to initial value, respectively. The effect of the group was non-significant (group contrast: control versus VSS: *t*(51.00) = −1.59, *P* = 0.12, *Cohen's d* = 0.28), indicating an absence of notable group differences in average GR power across trials 15–137.

We fitted the same model to the single-trial GR power estimated at the source level. The effects of trialN (*t*(20.17) = 5.56, *P* < 0.0001) and trialN × group interaction (*t*(51.15) = −2.30, *P* = 0.026) remain significant. This suggests that the steeper repetition-related increase of GR power observed in participants with VSS compared to controls at the sensor level was also present at the source level. Additionally, there was a trend towards a greater GR response in VSS than in controls across trials 15–137 (group contrast: control [group1] versus VSS: *t*(50.01) = −1.75, *P* = 0.09, *Cohen's d* = 0.28).

The individual regression coefficients (see Methods) were significantly different from zero in both groups (Mean_VSS_ = 0.246, SD_VSS_ = 0.160, *t*(25) = 7.84, *P* < 1e−6, *Cohen's d* = 1.57; Meam_Control_ = 0.168, SD_Control_ = 0.152, *t*(26) = 5.72, *P* < 0.00001, *Cohen's d* = 1.12) (Fig. 3C). Mann–Whitney U-test confirmed group differences in regression coefficients (*N*_VSS_ = 26, *N*_Control_ = 27, *Z* = 2.14, *P* = 0.03, *r* = 0.29, *Cohen's d* = 0.61), supporting the LMM finding of stronger repetition-related GR power increases in VSS participants. There was no correlation between individual slope coefficients of repetition-related GR power changes and visual discomfort scores in patients with VSS (*N* = 24, Spearman *R* = 0.027).

To gauge repetition-related changes of GR power across different drift rate conditions, we averaged GR power values in 41-trial blocks (15–55, 56–96, 97–137) according to stimulus type. Analysis of the medians of the averaged values showed that the GR power in the third block exceeded that in the first block for all drift rates and in both groups of participants (Fig. 3D). This trend was further supported by visual inspection of the GR power spectra, which were averaged across blocks and displayed separately for each condition and group (VSS versus control; see Supplementary Fig. 2).

To rule out baseline gamma power changes as a driver of group differences in stimulus repetition-related GR power increases, we applied the model: baseline_power ∼ trialN ∗ group + (1 + trialN ∣ subject). Results revealed no significant effect of group (*t*(51.00) = 0.19, *P* = 0.85) or trialN*group interaction (*t*(50.96) = 1.24, *P* = 0.22), indicating that the observed group difference in the repetition-related GR power increase is unlikely to be attributed to baseline changes.

In summary, both control and VSS participants exhibited a rapid decrease in GR power during the initial trials, followed by a sustained increase over the subsequent 100+ trials. Notably, VSS participants showed a more pronounced increase in GR power across trials. We will hereafter refer to the trial-by-trial changes in GR power as ‘repetition-related GR changes’.

### Stimulus repetition-related GR power increase is associated with HRV

No significant group differences were found in any of the HRV metrics during either rest or visual task (Supplementary Table 1). VSS group showed a trend towards a higher BPM compared to the control group during rest (*N*_VSS_ = 26, *N*_Control_ = 23, *Z* = 1.74, *P* = 0.08). We then investigated the relationship between individual GR power regression coefficients and HFnu, a measure that reflects the relative contribution of parasympathetic activity to autonomic balance.^51,52^

**Figure 4:**
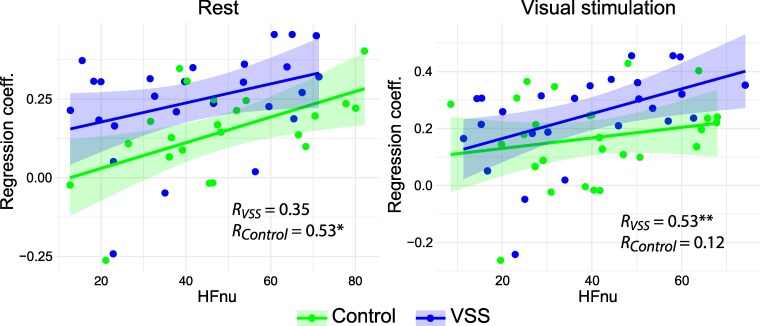
**Association between repetition-related gamma response (GR) changes and autonomic balance.** Regression coefficients quantify GR power changes across stimulus repetitions in patients with VSS and controls. Autonomic balance was estimated using normalized high-frequency heart rate variability (HFnu). Spearman's correlation coefficient shown as *R*; *P < 0.05, **P < 0.01.

During rest, GR power regression coefficients showed a significant correlation with HFnu in the overall sample (*N* = 49, *R* = 0.32, *P* = 0.03) and in the control group (*N* = 23, *R* = 0.53, *P* = 0.01). A similar trend was observed in the VSS group (*N* = 26, *R* = 0.35, *P* = 0.08). During visual stimulation, the correlation between GR power regression coefficients and HFnu remained significant in the combined participant sample (*N* = 53, *R* = 0.30, *P* = 0.03) and VSS (*N* = 26, *R* = 0.53, *P* = 0.006), but not the control group (*N* = 27, *R* = 0.12, *P* = 0.55). Figure 4 illustrates the relationship between GR power regression coefficients and HFnu. Correlations between GR power regression coefficients and other HRV metrics are detailed in Supplementary Table 2.

### Repetition-related increase in GR frequency does not differentiate between VSS and control groups

The model GR_frequency ∼ trialN + (1 + tria l N|subject) + (1 | condition)], provided a better fit to the GR power data than the more complex model that included the slope for the condition random factor. The effect of stimulus repetition within trials 15–137 was significant (*t*(51.28) = 2.64, *P* = 0.01) due to an increase in GR frequency with time. GR frequency increased for 0.43 Hz per 100 trials (CI_95%_ = [0.13 0.76]). The inclusion of the group factor in this model resulted in no significant effects for either the group (*t*(51.0) = 0.86, *P* = 0.39) or the trialN * group interaction (*t*(50.5) = 1.12, *P* = 0.27).

### Habituation of evoked responses does not differentiate between VSS and control groups

A detailed description of the ERF results is provided in the Supplementary materials, including Supplementary Fig. 3. Briefly, both control participants and individuals with VSS exhibited consistent habituation of ERF components (M80, M180). While no significant group differences in habituation were observed, there was a trend towards reduced M80 habituation in the VSS group.

## Discussion

In this MEG study, we investigated potential abnormalities in visual oscillatory gamma response (GR) parameters in patients with visual snow syndrome (VSS) that reflect the *E–I* balance and Hebbian neuroplasticity in the early visual cortex. Our results indicated no significant differences between VSS and control groups in modulation of GR power by varying visual stimulation intensity, suggesting an intact *E–I* ratio in VSS. In addition, GR power and frequency did not differ significantly between VSS participants and controls for any stimulus type. Both groups exhibited biphasic changes in GR power with stimulus repetition: an initial rapid decrease over the first few trials, followed by a gradual increase across approximately 100 subsequent repetitions. Notably, VSS patients demonstrated a significantly amplified GR power increase during the second phase. These findings imply that enhanced neuroplasticity in the early visual cortex may play a role in the pathophysiology of VSS, offering new insights into the underlying mechanisms of this condition. Therefore, in this section, we will first discuss the effect of stimulus repetition on GR power and explore its link to enhanced neuroplasticity in VSS. We will then examine potential reasons for the discrepancy between our findings and previously reported differences in the magnitude of the visual GR between VSS and control subjects.

The biphasic changes in single-trial visual GR power with stimulus repetition observed in our study (Fig. 3A) closely resemble those recently described for the first time in MEG of healthy human subjects.^36^ Stauch and colleagues demonstrated that repetitive presentation of an oblique grating initially led to a rapid decrease in visual GR power (time constant: 3.5 repetitions), followed by an approximately linear increase over the subsequent ∼100 repetitions. Importantly, these repetition-related power changes were stimulus-specific: altering the grating orientation disrupted the pattern. This stimulus specificity, coupled with the persistence of the effect over several minutes, provides strong evidence for the involvement of experience-dependent plasticity mechanisms in shaping gamma oscillatory responses to repeated stimuli.

In our study, the annular grating pattern remained constant, while its drift rate varied randomly across trials. This additional variability may explain the slower initial habituation of GR power observed in our study, with a time constant between 7 and 8 repetitions in both groups. The slope of the subsequent repetition-related increase in GR power was positive for nearly all participants (Fig. 3C), indicating a reliable repetition-related gamma increase. Consistent with previous findings,^36^ we also observed a repetition-related increase in GR frequency.

These repetition-related GR changes are thought to reflect Hebbian plasticity, characterized by strengthened synaptic connections among the ‘most responsive’ neurons and weakened connections among the ‘less responsive’ ones.^33,36^ Studies in monkeys using peripheral grating presentations demonstrated that the repetition-related increase in GR power was accompanied by an initial decrease and a subsequent stabilization in neuronal spiking activity.^33,55^ This pattern suggests that the observed GR power increase reflects enhanced neural synchronization rather than elevated neural excitation. Galuske *et al*.^38^ demonstrated in cats that an increase in gamma power induced by repeated exposure to a full-field grating with a specific orientation did not alter the firing rates of neurons tuned to that orientation. However, it did increase the activity of neurons tuned to close orientations.

Importantly, both described neuronal mechanisms suggest that stimulus repetition enhances the involvement of V1 neuronal populations in synchronous gamma oscillations, thereby strengthening their collective neural impact on postsynaptic target neurons in downstream cortical areas.^36,56-59^

From this perspective, the enhanced repetition-related gamma synchronization in V1 of patients with VSS may contribute to overstimulation of their downstream secondary and associative visual areas. Metabolic abnormalities in these regions, both during rest and visual stimulation, are consistently reported in this neurological disorder.^1,10,12^ Our results suggest that the observed hypermetabolism in higher-tier visual areas in VSS may thus be a consequence of the amplified input from V1, reflecting a cascade effect of altered neural processing throughout the visual hierarchy.

Regardless of precise mechanisms, the enhanced repetition-related changes in gamma response power observed in VSS participants likely reflect heightened Hebbian plasticity associated with activity-dependent modifications in neural representations of frequently encountered visual stimuli. Notably, animal studies have underscored the causal role of the maladaptive neural plasticity in the generation of tinnitus,^60^ an auditory misperception disorder often considered analogous to VSS (e.g.^27^). Given our findings, the high comorbidity of tinnitus in VSS patients suggests a shared pathophysiological mechanism involving altered neural plasticity.

The association between repetition-related gamma enhancement and the cardiac-derived index of autonomic balance provides additional indirect support for the ‘plasticity interpretation’ of our gamma findings. Our study revealed that a greater shift towards parasympathetic autonomic activity correlated with a steeper repetition-related increase in GR power (Fig. 4). States characterized by parasympathetic dominance appear to facilitate neuroplasticity and cortical remapping of representations of repeated stimuli. Indeed, numerous human and animal studies demonstrate that chronic or acute parasympathetic activation, such as through a vagus nerve stimulation, enhances stimulus-specific plasticity across sensory modalities by activating multiple neuromodulatory systems.^40,61-63^

While the observed correlation between the shift towards parasympathetic activity and repetition-related changes in GR power aligns with the role of functional states associated with parasympathetic activation (e.g. selective attention^64^) in promoting neuronal plasticity,^65^ we found no differences in HRV parameters between participants with VSS and controls. Thus, the observed group differences in the repetition-related GR power changes are unlikely to be attributed to differences in autonomic regulation.

Another potential factor could be a between-group difference in the pupillary response to visual stimuli. Stauch *et al*.^36^ reported a strong correlation between pupil constriction and gamma-band power suppression in neurotypical individuals during initial trials. They suggested this association likely reflects parallel adaptive processes, such as arousal and habituation, rather than a direct causal link. Since we did not record pupil size, we cannot exclude the possibility that group differences in pupillary constriction might have affected gamma oscillations and contributed to the observed differences between VSS and control participants. However, previous reports of intact pupil reactivity in VSS^66^ make this explanation unlikely.

Although our results suggest that aberrant neuroplasticity may play a role in the pathophysiology of VSS, we did not find a significant correlation between increased repetition-related gamma synchronization—a marker of enhanced plasticity—and the severity of visual discomfort in patients with VSS. This indicates that the observed neural changes in the early visual cortex, a primary generator of visual gamma oscillations,^17,18^ may not directly relate to other neurological alterations common in VSS patients. Similar dissociations were found in tinnitus, where self-reported distress correlates more with the activity of the stress-related brain regions and their connectivity to the auditory cortex rather than with sensory cortex activation.^67-69^ Further research is necessary to determine whether the enhanced plasticity in V1 observed in VSS patients predisposes them to the disorder's core symptom—phantom visual ‘snow’ sensations.

Another unresolved issue is the potential influence of migraine comorbidity on the observed differences in GR between patients with VSS and control participants. Migraine frequently co-occurs with VSS^70^ and is associated with distinct visual processing abnormalities, including photophobia, heightened sensitivity to patterned stimuli, and visual aura.^71-74^ Although our study was not designed to isolate migraine-specific effects, the high prevalence of migraine in our cohort (77%) suggests that comorbid pathophysiology may have contributed to the observed repetition-dependent gamma plasticity. Future studies comparing VSS patients with and without migraine, alongside migraineurs without VSS, are needed to clarify this issue.

Our findings regarding alterations in neuroplasticity in VSS may have clinical implications for therapeutic interventions for this disorder. They suggest that targeting neuroplasticity through neuropharmacological, instrumental, or psychotherapeutic approaches may be effective in alleviating both visual and psychological symptoms associated with this condition. Although evidence for the efficacy of such treatments in VSS remains limited, some intervention studies with diverse methodologies show promising results.^75-77^ For example, up to 20% of VSS patients report benefits from lamotrigine,^78,79^ an antiepileptic drug that not only reduces neuronal excitability by inhibiting glutamate release but also attenuates long-term potentiation (LTP) and long-term depression (LTD)-like synaptic plasticity.^75^ The neurofunctional indices of neuroplasticity identified in our study could aid in selecting patient populations most likely to benefit from targeted interventions and serve as biomarkers for monitoring treatment efficacy.

Despite group differences in repetition-related GR changes, the average power and frequency of GR induced by static or moving gratings, as well as their modulation by grating drift rate, remained comparable between groups (Fig. 2). These findings suggest that any increased neuronal excitability in VSS^6,7^ was not pronounced enough to alter the characteristics of non-invasively recorded gamma activity.

Notably, Hepschke *et al*. reported increased power of visually induced gamma oscillations in response to static stimuli in VSS patients compared to healthy controls.^14^ The authors interpreted this finding as indicative of increased excitability of V1. The discrepancy between our findings and those of Hepschke and colleagues may reflect the heterogeneity of VSS, where elevated gamma synchronization in the V1 might only occur in a subset of the patients, potentially leading to sampling bias.

A more critical point of debate, surpassing the discrepancy in findings discussed earlier, is whether absolute GR power recorded via MEG reliably reflects neuronal excitability. From a functional perspective, increases in GR power may signify enhanced neural synchronization rather than an actual rise in neuronal firing rates,^55^ which is the definitive indicator of neuronal excitability. Moreover, relying on GR power measured in a single experimental condition as an indicator of the excitability of an individual's visual cortex is particularly problematic. GR power is highly sensitive to stimulation parameters,^80^ and this sensitivity can vary considerably between subjects and clinical conditions, complicating direct comparisons. Within a group, for instance, a subject may exhibit relatively high GR power in one stimulation condition but low GR power in another.^80^

Assessing the *modulation* of GR parameters by excitatory drive may offers a more reliable approach for estimating neural excitability and *E–I* balance in the early visual cortex. A detailed discussion of this issue is available in our previous studies (e.g.^26^); here, we provide a brief summary. A gradual increase in excitatory drive to V1 results in a rapid rise in excitation and a slower, concurrent increase in inhibition.^81^ According to computational models, at high excitatory drive, when the inhibitory population of neurons is recruited faster than the excitatory population, the power of gamma oscillations begins to decrease.^24,25^ Consequently, the degree of GR power reduction when excitatory drive rises beyond the ‘optimal’ point for gamma generation may serve as a marker of neural inhibition efficiency in controlling the increasing excitation within V1 circuitry.

We have previously observed that the modulation of GR power by varying drift rates of a grating (a proxy for excitatory drive) is altered in conditions associated with *E–I* imbalance.^23,26^ For instance, children with autism characterized by inefficient inhibition in the V1^82^ exhibited reduced GR suppression at higher drift rates, indicating an elevated *E–I* ratio.^26^ In this context, the absence of abnormalities in GR power modulation among patients with VSS (Fig. 2A and B) suggests a preserved *E–I* ratio in their area V1.

Beyond GR power, GR frequency and its modulation by excitatory drive were also found to be normal in participants with VSS (Fig. 2C). Animal and computational studies consistently associate the modulation of gamma oscillation frequency (e.g. by stimulus contrast, velocity, or size) with the functionality of fast-spiking PV + interneurons.^21,83,84^ Previously, we documented attenuated GR frequency modulation in children with autism,^26^ consistent with findings from animal models indicating PV + interneuron dysfunction in the V1 as a characteristic feature of this condition.^82^ In contrast, the normal GR frequency and its modulation observed in VSS suggest that the functioning of this class of interneurons involved in gamma generation is preserved.

Taken together, our findings of relatively normal GR frequency and power modulation with increasing input drive in individuals with VSS suggest the absence of a significant inhibitory deficit or notable alterations in the *E–I* balance within the V1. It is likely, that the excitatory-inhibitory recurrent interactions responsible for generating and modulating gamma oscillations in V1 remain largely intact in VSS patients. Even if excitability is elevated, the *E–I* balance in the early visual cortex in individuals with VSS seems to remain well compensated, likely due to the preserved function of inhibitory neurons that effectively counteract increased excitation. This aligns with findings from Eren *et al*.,^8^ who reported no inhibitory deficits in the visual cortex of VSS patients in a TMS study. In this context, the heightened activity-dependent plasticity observed in VSS patients is unlikely to stem from impaired inhibition or an altered excitation-inhibition balance in the early visual cortex.

Another commonly observed effect of repeated visual stimuli, which we also found in this study, is the reduction in ERF/event-related potential (ERP) component amplitude, known as habituation. Mechanisms of ERF/ERP habituation are clearly different from those of repetition-related increase in the GR power and may include ‘fine-tuning’ of neurons and reducing cumulative cortical activation.^85^ While some previous studies observed attenuated ERP habituation in VSS^7,15,16^ (but see^86^), in our present study, the amplitudes of M80 and M180 ERF components demonstrated reliable habituation in both participants with VSS and controls. This habituation did not differ significantly between the groups. However, there was a trend towards reduced habituation of the early ‘M80’ component in VSS (see Supplementary materials for description and discussion). Future studies with larger sample sizes and optimized experimental methods are needed to explore potential differences in habituation between VSS and control individuals, as well as the relationship between the dynamics of repetition-related changes in ERP/ERF components’ amplitudes and GR power.

Our study has limitations. Our approach precluded analysis of how specific drift rates influenced trial-to-trial increases in gamma synchronization. Future studies employing a modified experimental paradigm, such as presentation of different drift rates in separate blocks, could address this question. Another limitation is the lack of eye-tracking during MEG recording: incorporating eye-tracking in future research will be important to test for the impact of pupil constriction and eye movements on our gamma findings. Lastly, our VSS participants represented a heterogeneous group with various comorbidities. Due to the limited sample size, we were unable to investigate the potential impact of these comorbidities on the MEG parameters analysed. Understanding how these factors influence repetition-related gamma synchronization in VSS is another important avenue for future research.

In conclusion, this study provides the first independent confirmation of the previously observed stimulus repetition-related increase in MEG-recorded visual gamma oscillations, which has been associated with neuroplasticity and perceptual learning in healthy individuals. Our key finding was that this repetition-related gamma increase is particularly pronounced in patients with VSS, suggesting heightened Hebbian neuroplasticity in this disorder. At the same time, the normal modulation of gamma power and frequency by the strength of excitatory input to the visual cortex in individuals with VSS suggests that this enhanced plasticity is unlikely to result from an altered *E–I* ratio or a deficiency of inhibitory neurons involved in the generation of gamma oscillations.

## Supplementary Material

fcaf505_Supplementary_Data

## Data Availability

The data tables and custom scripts used for statistical analysis and figure generation are deposited in a public repository and can be accessed via the permanent DOI: 10.48612/MSUPE/fb2u-9t6p-9vv7. The datasets analysed during the current study are not publicly available due to institutional data protection regulations. Access may be granted upon reasonable request and subject to approval by the institutional review board.
