# Supplementary material for: Enhanced neural plasticity of the primary visual cortex in visual snow syndrome: evidence from magnetoencephalographic gamma oscillations
Source: Brain Commun. 2025 Dec 24;8(1):fcaf505. doi: 10.1093/braincomms/fcaf505 (PMC12775836; doi:10.1093/braincomms/fcaf505)
Supplement: fcaf505_Supplementary_Data [file fcaf505_supplementary_data.docx]

**Enhanced Neural Plasticity of the Primary Visual Cortex in Visual Snow Syndrome: Evidence from MEG Gamma Oscillations**

Orekhova E.V.^1^*, Plieva, A.M.^1^, Naumova, S.M^1^, Obukhova T.S.^1^, Prokofyev A.O.^1^, Petrokovskaia А.V.^2^, Artemenko A.R.^3^, Stroganova T.A.^1^

^1^ Center for Neurocognitive Research (MEG Center), Moscow State University of Psychology and Education, Moscow, Russia

^2^ Loginov Moscow Clinical Scientific Center, Moscow, Russiа

^3^ Sechenov First Moscow State Medical University of the Ministry of Health of the Russian Federation (Sechenov University), Moscow, Russiа

**Supplementary Materials**

1. **Supplementary method: sensor-level analysis**

**1.1. MEG data preprocessing steps**

After processing with MaxFilter, the denoised MEG data were notch-filtered at 50:50:250 Hz. Independent Component Analysis (ICA) was then applied for biological artifact correction by detecting and removing independent components (ICs) correlated with heart activity and eye movements. The two groups did not differ in the mean number of ICs removed (N_VSS_=2.15; N_control_=2.29; Mann-Whitney U-test, Z=0.41, p=0.67). The MEG data were epoched from -1 to 1.2 s relative to stimulus onset. Epochs affected by bursts of myogenic activity or high-amplitude artifacts were identified first through automatic detection of high-amplitude signals (for magnetometers>4e-12T; for gradiometers>4000e-13T) and then through visual inspection, and excluded from the analysis.

**For all participants, data uninterrupted by breaks were available for the first 1 to 137 trials. The number of artefact-free trials did noth differ between groups for either the 1-137 trials interval (**mean N_Control_=132, mean N_vss_ = 131, Wilcoxon rank sum test: Z=1.69, p=0.09**) or for the whole experiment (**mean N_Control_=421, N_vss_ = 412, Z=1.40, p=0.17**). Excluded trials were approximately evenly distributed across time (Supplementary Figure 1).**

**Supplementary Figure 1.** Number of artifact-free trials included in the single-trial analysis, averaged across consecutive 41-trial blocks for both VSS and control groups. Median values are represented by bars, with whiskers indicating the range.

**1.2. Analysis of heart rate variability (HRV)**

HRV was assessed using the Systole v0.2.4 package^1^. Two periods of data were analyzed: (1) a 5-minute rest period with eyes open and (2) the first block of the experiment, before the break. ECG data were processed to identify R-peaks using a moving average algorithm. Artifacts such as missed/additional peaks or ectopic beats were corrected using the ‘correct_rr’ function. ECG parameters assessed included heart rate in beats per minute (BPM), as well as basic HRV parameters in the time and frequency domains. The latter included mean standard deviation of R-R intervals (SDNN), mean square of serial differences (RMSSD), percent of successive differences larger than 50 ms (pNN50), high-frequency (0.15-0.40 Hz) power of the R-R interval spectrum (HF), and HF power in normalized units: HFnu = HF/(HF + LF), where LF is the power in the low-frequency range (0.04 to 0.15 Hz).

**1.3. Analysis of event-related fields (ERF)**

To analyze the time courses of ERF component amplitudes, we first identified the time windows in which individual ERF components were observed. The signal was low-pass filtered at 40 Hz and averaged over all trials, separately for each subject, after which the root mean square (RMS) of the signal across all subject's gradiometers was calculated. RMS values were averaged over all participants so that VSS and control groups contributed equally to the mean. The mean RMS signal had two distinct peaks with maxima at 84 and 179 ms (M80 and M180) (**Supplementary Figure** 3A). Based on the latencies of these peaks, we used time windows of 60-100 ms and 150-240 ms to estimate individual peak latencies for M80 and M180. To this end, we found the gradiometer with the maximal absolute peak amplitude in corresponding time windows. In the maximal peak was negative, the signal was flipped. Single-trial amplitudes were calculated as averages over time windows defined as M80_latency_±15 ms and M180_latency_±30 ms. We used averaging in the time windows rather than peak detection in the single-trial data to ensure that the component amplitude was not systematically influenced by changes in the ongoing alpha oscillations, whose power increases with increasing time-on-task^2^.

The peak latencies of the M80 and M180 components averaged across conditions did not differ between subjects with VSS and controls (M80: Mean_VSS_ = 84 ± 8.8 ms, Mean_control_  = 84 ± 8.6 ms, t(52) = 0.02, p = 0.98; M180: Mean_VSS_ = 180 ± 13.8 ms, Mean_control_ = 179 ± 16.2 ms, t(52) = 0.15, p = 0.88). Since we were primarily interested in repetition-related changes in component amplitudes, and the amplitudes were averaged over fairly wide time windows, we did not account for possible differences in component latencies between conditions in this study.

**2. Supplementary results: sensor-level analysis**

**2.1. LMM analysis of single-trial GR power**

Previous studies have highlighted the stimulus dependency of repetition-related GR changes^3,4^. Although the visual stimulus in our study had the same shape and contrast across trials, its drift rate varied between trials. The limited amount of data prevented us from testing the effects of the trial number and drift rate repetition within a single model. To determine which factor better explained the time-dependent changes in GR power, we compared two LMMs:

Model1: GR_power ~ trialN + (1 + trialN|subject) + (1 + trialN|condition)

Model2: GR_power ~ driftrateN + (1 + driftrateN|subject) + (1 + driftrateN|condition)

Model1 examined the effect of overall trial number (15-137), while Model2 focused on drift-rate-specific repetition number within the same range. Model1 demonstrated superior fit (AIC=13611, BIC=13671, logLik=-6796.3) compared to Model2 (AIC=13674, BIC=13734, logLik=-6827.8), indicating that trial order number was a more effective predictor of z-transformed GR power than the repetitions of specific drift rate in our study design.

**2.2. Gamma-range power spectra averaged in blocks of trials**

To check whether the steeper repetition-related increase in GR power in patients with VSS compared to control participants - as revealed by LMM analysis - was also evident in the raw GR power spectra, we plotted the spectra averaged across blocks of trials. The habituation phase included trials 1-14 (see Fig. 3A,B in the main manuscript), while the facilitation phase comprised trials 15-55, 56-96, and 97-137. Spectra were plotted separately for each condition and group (VSS vs. Control; **Supplementary Figure** 2). The figure demonstrates that this LMM-derived effect is clearly visible in the raw spectra.


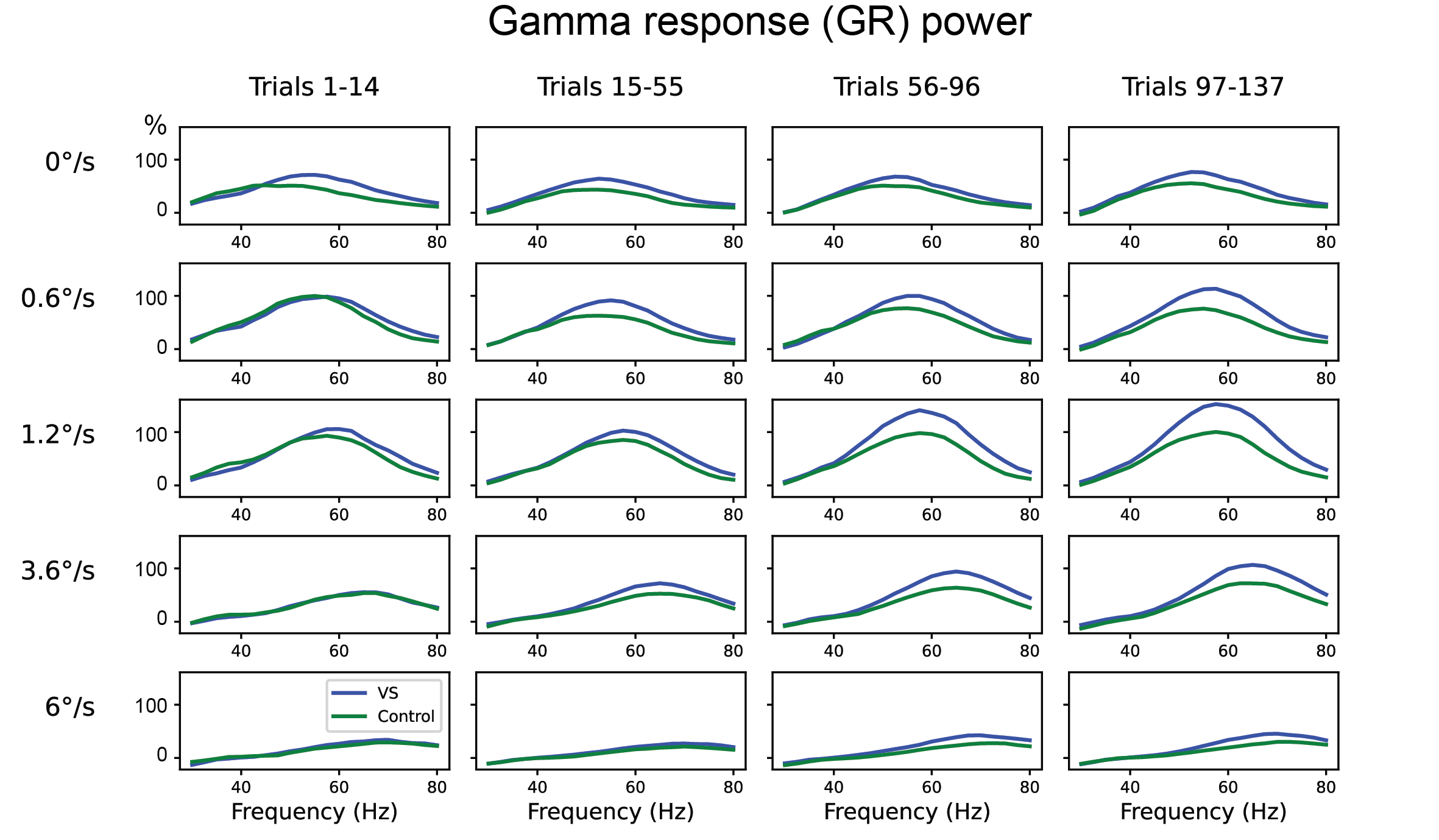


**Supplementary Figure 2.** Grand averaged Gamma Response (GR) power spectra – (stimulation-baseline)/baseline*100%–averaged in blocks of trials. Note that GR power increases from 15-55 trials block to 97-137 trials block, more so in patients with VSS than in controls.

**2.3. Heart Rate Variability (HRV)**

No significant group differences were found in any of the HRV metrics during either rest or during visual stimulation (Mann-Whitney U-test, all p>0.22; **Supplementary** Table 1).

**Supplementary Table 1.** Group comparison of the HRV metrics during rest with eyes open and visual stimulation.

| HRV Parameter | Rest | | | Visual stimulation | | |
| --- | --- | --- | --- | --- | --- | --- |
|  | Median, Control, N=22 | Median, VSS, N=26 | M-W U-test p-value | Median, Control N=27 | Median, VSS N=26 | M-W U-test p-value |
| RMSSD | 35.59 | 31.4 | 0.55 | 34.1 | 34.1 | 0.67 |
| SDNN | 51.3 | 49.8 | 0.30 | 48.9 | 52.5 | 0.64 |
| pnn50 | 15.5 | 8.5 | 0.70 | 13.1 | 12.9 | 0.77 |
| LF | 526 | 594 | 0.94 | 737 | 728 | 0.61 |
| HF | 577 | 381 | 0.23 | 501 | 531 | 0.78 |
| HFnu | 46.4 | 40.7 | 0.29 | 40.0 | 37.9 | 0.62 |

**Supplementary Table 2.** Spearman correlations between Gamma Response (GR) power regression coefficients and Heart Rate Variability (HRV) metrics in the combined sample of participants (Control + VSS).

|  | Rest (N = 48) | Visual stimulation (N = 52) |
| --- | --- | --- |
| RMSSD | 0.2 | 0.16 |
| SDNN | 0.05 | -0.03 |
| pnn50 | 0.18 | 0.18 |
| HF | 0.19 | 0.15 |
| LF | -0.11 | -0.15 |
| **HFnu** | **0.32*** | **0.30*** |

* p < 0.05

**2.4. Habituation of evoked responses does not differentiate between VSS and control groups**

First, we examined potential group differences in RMS values of the averaged signal within the M80 and M180 time windows (M80 latency ± 15 ms and M180 latency ± 30 ms) using a mixed ANOVA with factors of Group and Condition. No significant effects of Group or Group × Condition interaction were found for either component (all p > 0.3). However, the effect of Condition was significant for M80 (F(4, 204) = 3.9, G-G ε = 0.93, p = 0.004, ηp² = 0.07), which showed a decrease with increasing drift rate, and for M180 (F(4, 204) = 13.8, G-G ε = 0.49, p = 0.000006, ηp² = 0.21), which exhibited an increase at higher drift rates (**Supplementary Figure** 3B,C).

To analyze the temporal dynamics of M80 and M180 responses across the entire sample of participants, we employed a method similar to that used for GR parameters. First, amplitude values were z-transformed separately for each subject and condition, then averaged across all conditions and participants for visual inspection. The z-scaled M80 amplitudes showed a clear decreasing trend with increasing number of trials, reaching a plateau around the 60th sample (**Supplementary Figure** 3D). To identify the inflection point of this curve, we fitted a split line to the data, excluding the first trial due to its anomalously low M80 z-scored amplitude (**Supplementary Figure** 3D). This analysis pinpointed the inflection point at the 57th trial. Based on this finding, we applied a LMM to the single-trial M80 amplitudes for trials 2 - 57. This model accounted for slope and intercept for individual subjects and intercept for condition: [M80ampl ~ trialN + (1 + trialN | subject) + (1 | condition)]. The results revealed a significant effect of stimulus repetition (t(52.01) = 3.43, p = 0.001), indicating robust habituation across the sample. To investigate potential group differences, we expanded the model to include a fixed effect of group: [ M80ampl ~ trialN * group + (1 + trialN | subject) + (1 | condition) ]. There was a trend towards less pronounced M80 amplitude habituation in the VSS group (t(51.17) = 1.70, p = 0.096). This subtle difference is illustrated in **Supplementary Figure** 3E using z-scored data.


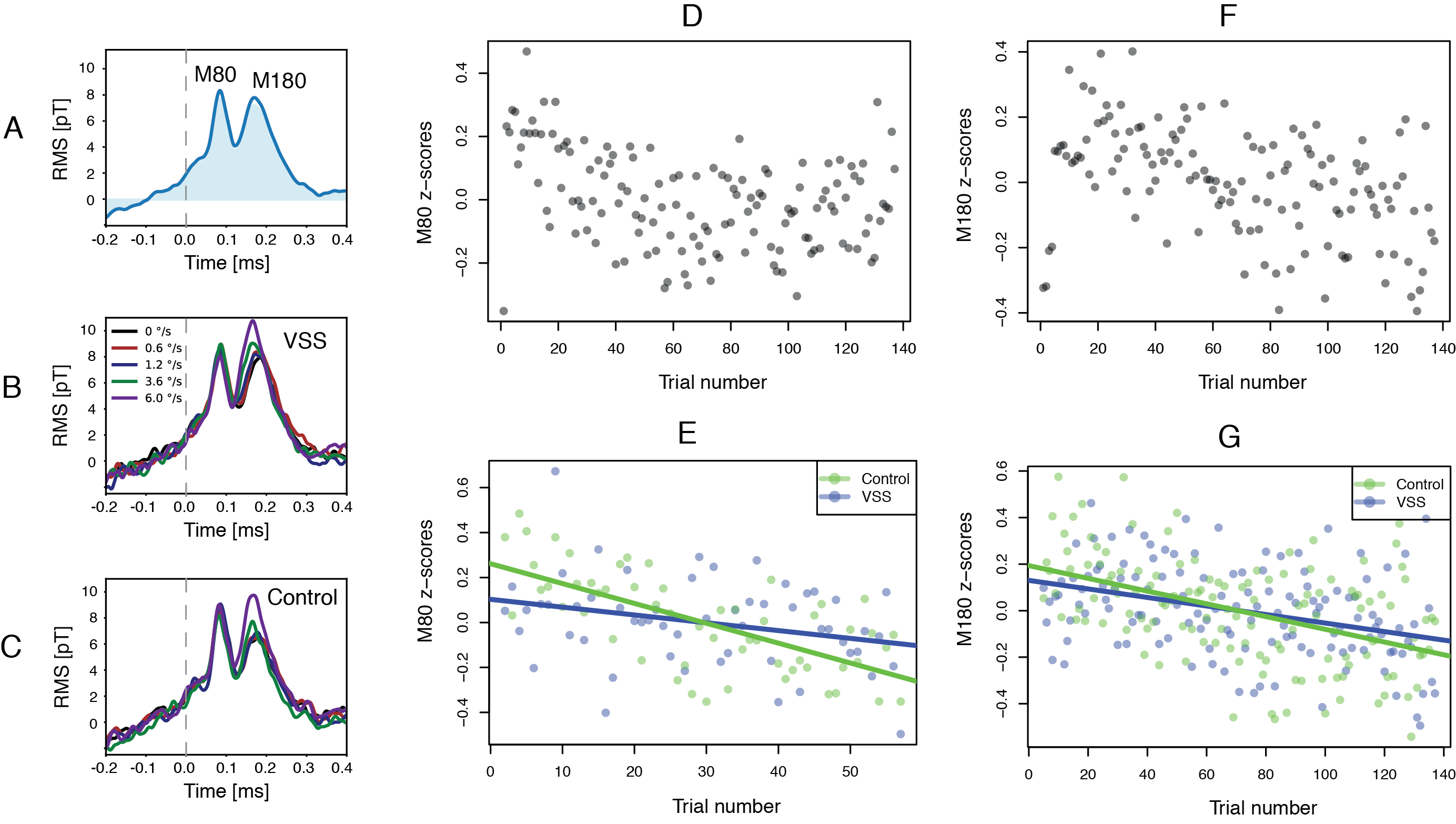


**Supplementary Figure** **3.** Changes in amplitude of event-related fields (ERF) associated with stimulus repetition. **A.** Root mean squared (RMS) signal averaged over all gradiometers, participants and drift rate conditions. **B,C.** RMS signals averaged separately for drift rate conditions, for VSS and control participants, respectively. **D.** Time course of z-scored M80 amplitude across 1 - 137 trials in the combined sample of participants. **E.** Time courses of z-scored M80 amplitude in VSS and control participants across 2 - 57 trials. Linear mixed model (LMM) analysis indicated significant M80 habituation across all participants (p = 0.001), with a non-significant trend for reduced habituation in the VSS group (p = 0.096). **F.** Time course of z-scored M180 amplitude across 1 - 137 trials in the combined sample of participants. **G.** Time courses of z-scored M180 amplitude in VSS and control participants across 5-137 trials. LMM analysis confirmed significant M180 habituation across participants (p = 0.001), with no group difference (p = 0.31).

The z-scored M180 amplitudes exhibited a nearly linear decrease with increasing trial order number, with a notable exception for the first four trials, which deviated from this habituation pattern (**Supplementary Figure** 3F). To account for this initial irregularity, we excluded these four trials from the subsequent analysis. We then applied the LMM to the single-trial M180 amplitudes for trials 5-137, using the following model: [M180ampl ~ trialN * group + (1 + trialN | subject) + (1 | condition)]. This analysis revealed a significant effect of trial order number (t(51.89) = 3.36, p = 0.001), indicating a robust habituation effect across the combined sample of participants. The interaction between a trial order number and group was not significant (t(50.95) = 1.03, p = 0.31). These results, illustrated in **Supplementary Figure** 3G using z-scored data, suggest that while there is a clear habituation effect in M180 amplitude across all participants, this effect does not significantly differ between the VSS and control groups.

In conclusion, our analysis revealed that neither M80 nor M180 amplitude habituation significantly differentiated between VSS and control groups. However, a subtle trend towards reduced M80 amplitude habituation was observed in VSS participants, suggesting a potential difference in neural adaptation processes.

**2.5. The relationship between repetition-related changes in GR power and EFR components**

To investigate a potential link between the habituation of evoked components and stimulus repetition-related changes in GR power, we first quantified these changes in M80 and M180 using the same approach as for GR power. Specifically, we fitted linear regressions to the z-transformed amplitude values for each drift rate condition [M80amplitude ~ trialN, M180amplitude ~ trialN] and then averaged the linear regression coefficients across conditions for each subject.

The coefficients were significantly different from zero for both VSS and control participants in the M80 component (VSS: mean = -0.075, SD = 0.156, t(25) = 2.46, p = 0.02; Control: mean = -0.170, SD = 0.200, t(26) = 4.41, p = 0.0002) as well as in the M180 component (VSS: mean = -0.075, SD = 0.168, t(25) = 2.27, p = 0.03; Control: mean = -0.116, SD = 0.190, t(26) = 3.17, p = 0.004).

In the control group, the correlations between evoked response regression coefficients and GR power regression coefficients were not significant (N = 27; M80: R = -0.02, M180: R = 0.07). The VSS group showed a trend towards a positive correlation between M80 and gamma power regression coefficients (N = 26; R = 0.35, p = 0.08), suggesting a potential link between weaker M80 habituation and stronger gamma increases in VSS participants. The M180 regression coefficient did not correlate with the gamma slope in the VSS (N = 26; R = -0.02, n.s.) or control (N = 27; R = 0.07, n.s.) groups.

**3. Supplementary discussion: sensor-level analysis**

A question arising from our results is the comparative sensitivity of two neurophysiological indices—facilitation of visually induced gamma oscillations and habituation of phase-locked evoked responses (ERF/ERP)—to potential neuroplasticity abnormalities in the early visual cortex in VSS. In our study, repeated exposure to the stimulus affected both the induced gamma oscillations (Figure 3 in the main manuscript) and the magnitude of visual evoked responses (**Supplementary Figure** 3E,G). ERP/ERF habituation, defined as a decrease in component amplitude upon repetition, is thought to reflect the ‘fine-tuning’ of neurons, optimizing responses to repetitive stimuli and reducing cumulative cortical activation^5^. Previous studies have frequently reported a lack of visual ERP habituation in migraine, a condition often comorbid with HRV, during the interictal period^6^, but see^7^. Although similar trends have been reported in a few VSS studies^8-10^, limited results remain inconsistent^11^.

In the present study, we observed reliable habituation of visual ERF components (M80 and M180) in both control participants and those with VSS (**Supplementary Figure** 3E,G). This result resembles the findings of Stauch et al.^4^, who presented healthy participants with static gratings for 0.3 - 2 seconds with 1-second intervals and described a trial-dependent decrease in the magnitude of the ‘early’ and ‘late’ ERF components induced by the gratings (55 - 70 and 90 - 180 ms, respectively, in their study). ERF activity around ~70 - 80 ms is likely, at least partially, generated in the early visual cortex^12^, and the common origin in V1 makes it possible to compare its repetition-related changes with those observed in GR power. In our study, the magnitude of the M80 peak gradually decreased during the first ~60 stimulus repetitions and then reached a plateau (**Supplementary Figure** 3D). This time course contrasts with the dynamics of GR power, which decreased rapidly during the first few presentations and then gradually increased over at least 100 trials (Figure 3A in the main manuscript). VSS patients exhibit a trend toward attenuated M80 habituation, along with a tendency for correlation between reduced M80 habituation and increased GR facilitation during the later phase of stimulus repetitions. Although both M80 findings did not reach statistical significance, we report them to emphasize the need for future studies with larger sample sizes and more refined experimental designs to clarify the relationship between ERF habituation and gamma amplification in VSS. However, given the different trial-wise dynamics of these electrophysiological measures, they are likely driven by separate, co-occurring repetition-related mechanisms within the early visual cortex. Notably, only gamma synchronization exhibited atypical repetition-related patterns in VSS in the present study.

**4. Supplementary method: source-level analysis**

**4.1. Structural MRI**

Structural MRIs were available for 26 of 27 control participants and for 20 of 26 participants with VSS. When available, structural MRI data were acquired using 1.5 T or 3 T scanners. T1-weighted images with voxel size 1mm^3^ were preprocessed and processed with the default algorithm (‘recon-all’) implemented in FreeSurfer software (v.6.0.0). This algorithm reconstructs a two-dimensional cortical surface from a three-dimensional volume. It removes the skull from the brain volume, creates a brain mask, segments the volume into cortex, whitematter, and subcortical structures, and finds white and gray matter boundaries (see https://surfer.nmr.mgh.harvard.edu/fswiki/recon-all for a detailed description of the processing steps). The cortical white/gray matter boundary was further used to create individual surface-based source models for distributed source localization.

**4.2. MEG source localization**

MEG data were co-registered with the structural MRI of the head, and a single-layer boundary element model was constructed. In case the individual MRI was not available, we applied a recently developed ‘pseudo-MRI’ method ^13^ to co-register the template MRI (MNI152 linear template; https://www.bic.mni.mcgill.ca/ServicesAtlases/ICBM152NLin2009) with the subject’s headshape obtained during MEG experiment and to create subject-speciphic pseudo-MRI. We then created surface-based source space with 4096 vertex sources in each hemisphere and estimated the forward solution.

The raw data were down-sampled to 500 Hz and filtered using 30 Hz high-pass and 115 Hz low-pass finite impulse response filters with default parameters. Noise covariance matrices and data covariance matrices were estimated based on the prestimulus (-1–0 s, relative to the stimulus onset) and stimulation (0–1.2 s, relative to the stimulus onset) time intervals, respectively, of all conditions combined. Linearly constrained minimum variance (LCMV, ^14^) beamformer spatial filters with a source orientation that maximizes power and a regularization coefficient 0.05 were created separately for each stimulus type and were applied individually to each epoch. As we expected to observe gamma oscillations in the visual cortex, the inverse solution was limited to visual and adjacent cortical areas, same as in ^15^.

**4.3. Analysis of gamma oscillations in source space.**

The approach to the time-frequency analysis of gamma oscillations in source space mirrored that in sensor space. The main difference was that instead of selecting ‘maximal sensors’, for each participant, among all the five conditions, we have chosen 26 vertices with maximum response power in gamma range (40–80 Hz), and averaged the spectra over these vertices, separately for each condition.

**5. Supplementary results: source-level analysis**

**5.1. Localization of gamma response.**

**Supplementary Figure** 4 shows gamma responses averaged in each group, separately for each of five drift rate conditions.


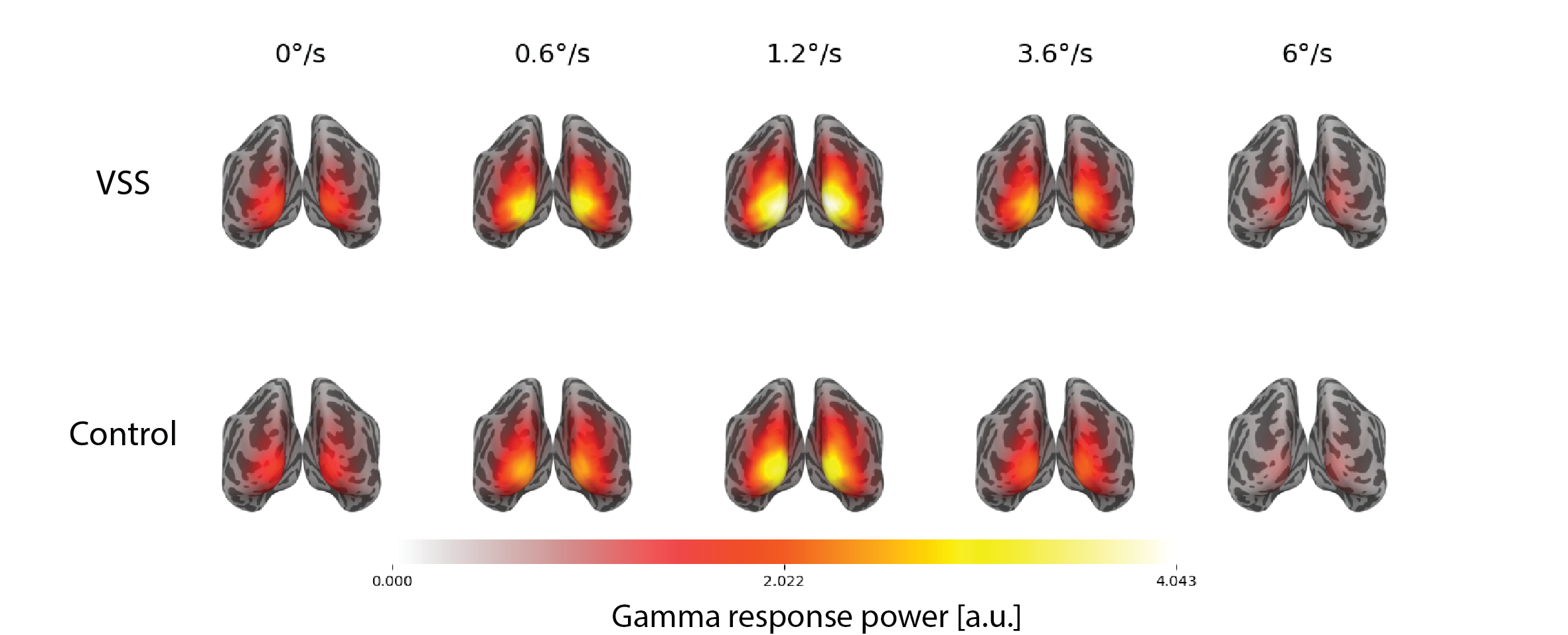


**Supplementary Figure 4.** Source localization of gamma response (GR) in participants with VSS and control subjects: group average. The scale represents GR power calculated as (stimulation-baseline)/baseline.

**Supplementary** Table 3 presents the MNI coordinates of the ‘maximal gamma sources’ for each group and condition. These sources were identified based on grand-average data (**Supplementary** Figure 4) as the dipole locations exhibiting the highest GR power. In all cases and across both hemispheres, the ‘maximal gamma sources’ were localized within the primary visual cortex.

**Supplementary Table 3**. Cortical localization of the maximal GR sources.

| **group** | **condition** | **Hemi-sphere** | **mni_x** | **mni_y** | **mni_z** | **label** |
| --- | --- | --- | --- | --- | --- | --- |
| Control | static | lh | -7.87542963 | -99.64810944 | 5.25385046 | Primary Visual Cortex (V1)-lh* |
| Control | static | rh | 15.19758797 | -100.9972305 | 0.885055363 | Primary Visual Cortex (V1)-rh |
| Control | 0.6 °/s | lh | -11.80121517 | -99.98722076 | -0.705916882 | Primary Visual Cortex (V1)-lh |
| Control | 0.6 °/s | rh | 15.19758797 | -100.9972305 | 0.885055363 | Primary Visual Cortex (V1)-rh |
| Control | 1.2 °/s | lh | -7.87542963 | -99.64810944 | 5.25385046 | Primary Visual Cortex (V1)-lh |
| Control | 1.2 °/s | rh | 11.49874496 | -91.54664612 | -2.953929901 | Primary Visual Cortex (V1)-rh |
| Control | 3.6 °/s | lh | -6.675663948 | -93.09111023 | 8.113061905 | Primary Visual Cortex (V1)-lh |
| Control | 3.6 °/s | rh | 11.49874496 | -91.54664612 | -2.953929901 | Primary Visual Cortex (V1)-rh |
| Control | 6 °/s | lh | -11.80121517 | -99.98722076 | -0.705916882 | Primary Visual Cortex (V1)-lh |
| Control | 6 °/s | rh | 11.49874496 | -91.54664612 | -2.953929901 | Primary Visual Cortex (V1)-rh |
| VSS | static | lh | -7.87542963 | -99.64810944 | 5.25385046 | Primary Visual Cortex (V1)-lh |
| VSS | static | rh | 12.19224358 | -94.24595642 | 8.42461586 | Primary Visual Cortex (V1)-rh |
| VSS | 0.6 °/s | lh | -7.87542963 | -99.64810944 | 5.25385046 | Primary Visual Cortex (V1)-lh |
| VSS | 0.6 °/s | rh | 12.19224358 | -94.24595642 | 8.42461586 | Primary Visual Cortex (V1)-rh |
| VSS | 1.2 °/s | lh | -7.87542963 | -99.64810944 | 5.25385046 | Primary Visual Cortex (V1)-lh |
| VSS | 1.2 °/s | rh | 12.19224358 | -94.24595642 | 8.42461586 | Primary Visual Cortex (V1)-rh |
| VSS | 3.6 °/s | lh | -11.86039543 | -95.5606842 | 4.667784691 | Primary Visual Cortex (V1)-lh |
| VSS | 3.6 °/s | rh | 5.935656548 | -89.90046692 | 7.953664303 | Primary Visual Cortex (V1)-rh |
| VSS | 6 °/s | lh | -11.38275623 | -92.65003967 | 6.349103928 | Primary Visual Cortex (V1)-lh |
| VSS | 6 °/s | rh | 5.935656548 | -89.90046692 | 7.953664303 | Primary Visual Cortex (V1)-rh |

*According to the atlas HCPMMP1_combined (Glasser et al, Nature, 2016.)

**5.2. Statistical analysis of the gamma response in source space.**

For the source analysis of GR power we used the same LMM model and ANOVA design as for the sensor-level analysis.

To test for the group differences in the repetition-related increase in GR power from trial 15 to 137 (see Figure 3A,B in the main manuscript) we fitted the following LMM to the data:

GR_power ~ trialN * group + (1 + trialN|subject) + (1 + trialN|condition), where:

**trialN** is the trial order number (continuous predictor),

**group** is a categorical predictor with two levels: **VSS (first group)** and **control (second group)**,

**subject** and **condition** are included as random effects with random slopes for **trialN**.

LMM analysis revealed main effect of trial order number (trialN: T(20.17) = 5.56, p = 1.85e-5) and significant trialN × Group interaction (T(51.10) = -2.30, p = 0.026). In patients with VSS, GR power increased by 0.78 units per 100 trials (CI_95%_ = [0.52 1.05]), while in control participants the increase was 0.21 units (CI_95%_ = [0.07 0.35]). Note that these values represent 78% and 21% increase relative to baseline, respectively. These results suggest that the steeper repetition-related increase in GR power observed in the sensor-space is also found in the source space. There was also a tendency for greater GR response in VSS than in control participants during these 15 to 137 trials (group2 (control): t(50.01) = -1.75, p = 0.09, *Cohen's d* = 0.28).

To quantify individual changes in gamma response (GR) power across trials 15–137, we next fitted a linear regression model (**z_score ~ trialN**), as in the sensor-space analysis. A follow-up t-test revealed a statistical trend toward a steeper increase in GR power in patients with visual snow syndrome (VSS) compared to control participants (p = 0.09).

For the averaged GR estimated over all available trials (1 to 450), the results of the mixed ANOVAs were also similar to those obtained in the sensor space.

For GR power:

Group: F(1,50) = 0.57, p=0.45, ηp²=0.01;

Condition: F(4,200) = 73.9, G-G epsilon = 0.44, p<0.0001), ηp²=0.6;

Group * Condition: F(4,200) = 0.75, G-G epsilon = 0.44, p=0.46), ηp²=0.01;

For GR frequency:

Age: F(1,50) = 3.96, p=0.05, ηp²=0.07;

Group: F(1,50) = 0.23, p=0.63, ηp²=0.00;

Condition: F(4,200) = 280.0, G-G epsilon = 0.38, p<0.0001), ηp²=0.85;

Group * Condition: F(4,200) = 0.15, G-G epsilon = 0.37, p=0.96), ηp²=0.00;

**Supplementary** **references**

1. Legrand N, Allen M. Systole: A python package for cardiac signal synchrony and analysis. *J Open Source Software*. 2022;7(69):3832. doi:10.21105/joss.03832

2. Benwell CSY, London RE, Tagliabue CF*, et al*. Frequency and power of human alpha oscillations drift systematically with time-on-task. *NeuroImage*. May 15 2019;192:101-114. doi:10.1016/j.neuroimage.2019.02.067

3. Peter A, Stauch BJ, Shapcott K*, et al*. Stimulus-specific plasticity of macaque V1 spike rates and gamma. *Cell reports*. Dec 7 2021;37(10):110086. doi:10.1016/j.celrep.2021.110086

4. Stauch BJ, Peter A, Schuler H, Fries P. Stimulus-specific plasticity in human visual gamma-band activity and functional connectivity. *eLife*. Aug 24 2021;10. doi:10.7554/eLife.68240

5. Grill-Spector K, Henson R, Martin A. Repetition and the brain: neural models of stimulus-specific effects. *Trends in cognitive sciences*. Jan 2006;10(1):14-23. doi:10.1016/j.tics.2005.11.006

6. Puledda F, Ffytche D, Lythgoe DJ*, et al*. Insular and occipital changes in visual snow syndrome: a BOLD fMRI and MRS study. *Annals of clinical and translational neurology*. Mar 2020;7(3):296-306. doi:10.1002/acn3.50986

7. Marti-Marca A, Vilà-Balló A, Cerda-Company X*, et al*. Exploring sensory sensitivity, cortical excitability, and habituation in episodic migraine, as a function of age and disease severity, using the pattern-reversal task. *The journal of headache and pain*. Aug 7 2023;24(1):104. doi:10.1186/s10194-023-01618-w

8. Luna S, Lai D, Harris A. Antagonistic Relationship Between VEP Potentiation and Gamma Power in Visual Snow Syndrome. *Headache*. Jan 2018;58(1):138-144. doi:10.1111/head.13231

9. Unal-Cevik I, Yildiz FG. Visual Snow in Migraine With Aura: Further Characterization by Brain Imaging, Electrophysiology, and Treatment--Case Report. *Headache*. Nov-Dec 2015;55(10):1436-41. doi:10.1111/head.12628

10. Yildiz FG, Turkyilmaz U, Unal-Cevik I. The Clinical Characteristics and Neurophysiological Assessments of the Occipital Cortex in Visual Snow Syndrome With or Without Migraine. *Headache*. Apr 2019;59(4):484-494. doi:10.1111/head.13494

11. Eren O, Rauschel V, Ruscheweyh R, Straube A, Schankin CJ. Evidence of dysfunction in the visual association cortex in visual snow syndrome. *Annals of neurology*. Dec 2018;84(6):946-949. doi:10.1002/ana.25372

12. Tzelepi A, Ioannides AA, Poghosyan V. Early (N70m) neuromagnetic signal topography and striate and extrastriate generators following pattern onset quadrant stimulation. *NeuroImage*. Apr 2001;13(4):702-18. doi:10.1006/nimg.2000.0735

13. Jaiswal A, Nenonen J, Parkkonen L. Pseudo-MRI Engine for MRI-Free Electromagnetic Source Imaging. *Human brain mapping*. Feb 1 2025;46(2):e70148. doi:10.1002/hbm.70148

14. Van Veen BD, van Drongelen W, Yuchtman M, Suzuki A. Localization of brain electrical activity via linearly constrained minimum variance spatial filtering. *IEEE transactions on bio-medical engineering*. Sep 1997;44(9):867-80. doi:10.1109/10.623056

15. Manyukhina VO, Rostovtseva EN, Prokofyev AO*, et al*. Visual gamma oscillations predict sensory sensitivity in females as they do in males. *Scientific reports*. Jun 8 2021;11(1):12013. doi:10.1038/s41598-021-91381-2
